# Epidemiology of tuberculosis in the Western Pacific Region: Progress towards the 2020 milestones of the End TB Strategy

**DOI:** 10.5365/wpsar.2020.11.3.002

**Published:** 2020-12-28

**Authors:** Fukushi Morishita, Kerri Viney, Chris Lowbridge, Hend Elsayed, Kyung Hyun Oh, Kalpeshsinh Rahevar, Ben J Marais, Tauhid Islam

**Affiliations:** a End TB and Leprosy Unit, World Health Organization Regional Office for the Western Pacific, Manila, Philippines.; b Prevention, Diagnosis, Treatment, Care and Innovation Unit, Global TB Programme, World Health Organization, Geneva, Switzerland.; c Centre for Research Excellence in Tuberculosis and the Marie Bashir Institute for Infectious Diseases and Biosecurity, University of Sydney, Sydney, Australia.; d Menzies School of Health Research, Charles Darwin University, Darwin, Australia.

## Abstract

Since 2015, the End TB Strategy and the Regional Framework for Action on Implementation of the End TB Strategy in the Western Pacific 2016–2020 have guided national tuberculosis (TB) responses in countries and areas of the Region. This paper provides an overview of the TB epidemiological situation in the Western Pacific Region and of progress towards the 2020 milestones of the Strategy. A descriptive analysis was conducted of TB surveillance and programme data reported to WHO and estimates of the TB burden generated by WHO for the period 2000–2018. An estimated 1.8 million people developed TB and 90 000 people died from it in the Region in 2018. Since 2015, the estimated TB incidence rate and the estimated number of TB deaths in the Region decreased by 3% and 10%, with annual reduction rates of 1.0% and 3.4%, respectively. With current efforts, the Region is unlikely to achieve the 2020 milestones and other targets of the Strategy. Major challenges include: (1) wide variation in the geographical distribution and rate of TB incidence among countries; (2) a substantial proportion (23%) of TB cases that remain unreached, undiagnosed or unreported; (3) insufficient coverage of drug susceptibility testing (51%) for bacteriologically confirmed cases and limited use of WHO-recommended rapid diagnostics (11 countries reported < 60% coverage); (4) suboptimal treatment outcomes of TB (60% of countries reported < 85% success), of TB/HIV co-infection (79%) and of multidrug- or rifampicin-resistant TB (59%); (5) limited coverage of TB preventive treatment among people living with HIV (39%) and child contacts (12%); and (6) substantial proportions (35–70%) of TB-affected families facing catastrophic costs. For the Region to stay on track to achieve the End TB Strategy targets, an accelerated multisectoral response to TB is required in every country.

Tuberculosis (TB) continues to be a global public health problem, which disproportionately affects poor and marginalized populations who often have limited health care access. Despite the continued global effort to end TB, the disease continues to be the leading infectious killer on the planet. In 2018, an estimated 10 million people fell ill with TB and 1.5 million died from the disease. (*1*) Geographically, over 85% of TB cases in 2018 occurred in three WHO regions: 44% in South-East Asia, 24% in Africa and 18% in the Western Pacific. (*1*) In total, 30 countries with high burdens of TB accounted for 87% of the world’s cases. (*1*)

In 2014, the Sixty-seventh World Health Assembly endorsed a global strategy, now commonly known as the End TB Strategy, (*2*) and targets for TB prevention, care and control after 2015. The Strategy set the ambitious target of ending TB by 2035, by reducing the incidence rate by 90% and the number of deaths by 95% from those in 2015. Interim 2020 milestones were defined as reductions of 20% of the incidence rate and 35% in the number of deaths, in addition to eliminating catastrophic costs incurred by TB. (*2*) Since 2014, two global high-level meetings (the WHO Global Ministerial Conference on “ending TB in the sustainable development era,” held in Moscow, Russian Federation, in 2017, and the first high-level meeting on TB at the United Nations General Assembly, in New York in 2018) created unprecedented political momentum to accelerate the global TB response. (*3*) The world is, however, unlikely to achieve the 2020 milestones, with only a 6.3% reduction in TB incidence and a 5.2% reduction in TB deaths reported between 2015 and 2018. (*4*) In addition, there is grave concern that the COVID-19 pandemic will set back the modest gains made to date. (*5*)

The WHO Western Pacific Region is home to  1.9 billion people in 37 countries and areas. The Region is diverse, comprising large countries with populations of more than 1 billion people and small Pacific island countries with a few thousand residents, as well as countries with high and intermediate TB burdens and others in the pre-elimination stage. The Regional Framework for Action on Implementation of the End TB Strategy in the Western Pacific 2016–2020 has guided national TB responses in countries and areas of the Region by proposing actions for national TB programmes. (*6*, *7*) This paper provides an overview of the epidemiology of TB in the Region and of progress towards the 2020 interim milestones of the End TB Strategy and the Regional Framework.

## Methods

We conducted a descriptive analysis of TB surveillance and programme data reported by countries and areas to WHO, and TB burden estimates generated by WHO for the Western Pacific Region, for the period 2000–2018. Countries and areas report data on TB to WHO annually via an electronic platform. The data are then verified and published in WHO’s Global TB Reports, in which WHO-generated estimates are also published. A full description of WHO’s data collection methods is available in the Global TB Report 2019; (*1*) the methods used to estimate TB incidence and mortality are provided in an online technical appendix. (*8*) We used the definitions of cases and treatment outcome given in the WHO reporting framework for TB. (*8*) All data sets are available from the WHO global TB database. (*4*) In 2019, data for 2018 were reported to WHO by 35 countries and areas in the Region, accounting for 99.9% of the regional population.

We reviewed trends in TB incidence and mortality, case notifications, indicators of collaborative TB/HIV activities and treatment outcomes. In addition to regional analyses, we also reviewed national data from the seven countries with high burdens of TB in the Region (Cambodia, China, the Lao People's Democratic Republic, Mongolia, Papua New Guinea, the Philippines and Viet Nam), which have more than 95% of the Region’s cases.

The cascade of care for TB, drug-resistant TB (DR-TB) and TB/HIV co-infection was assessed to identify and quantify gaps in care delivery. For DR-TB, the estimated number of incident multidrug- or rifampicin-resistant TB (MDR/RR-TB) cases, the case detection rate (the number of laboratory-confirmed MDR/RR-TB cases divided by the estimated number of incident cases) and the treatment enrolment rate (the number of cases enrolled in second-line treatment divided by the number of laboratory-confirmed cases) were provided for countries with the highest estimated numbers of MDR/RR-TB incidence. Indicators of TB prevention and catastrophic costs due to TB were assessed where data were available. Catastrophic costs for TB-affected families are defined as total costs (comprising direct medical and non-medical costs plus income losses) that represent 20% or more of annual household income. We also developed a colour-coded scorecard to summarize indicators of implementation of the End TB Strategy for each country in the Region. In this paper, “range” refers to the 95% uncertainty interval. All analyses were conducted with the statistical software package R 3.6.1 (Comprehensive R Archive Network at https://cran.r-project.org/).

Ethical clearance was not required, as this report was part of a regular evaluation of programme performance.

## Results

### Estimates of TB burden

**Figure 1:**
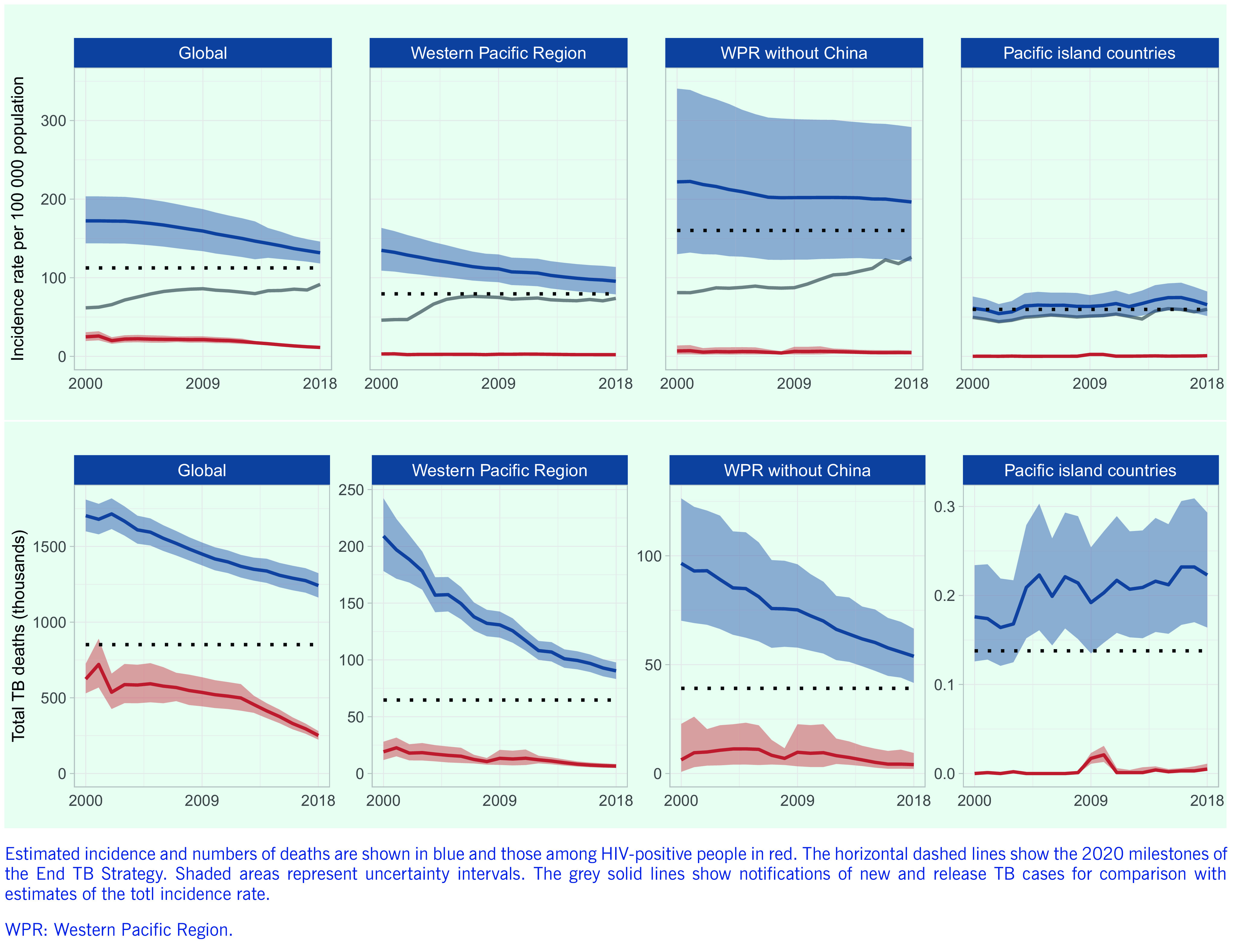
Trends in estimated TB incidence and total TB deaths at global and regional levels, 2000–2018

Since 2000, the estimated incidence of TB in the Region has decreased steadily, from 135 (range, 109–163) per 100 000 population (2.3 [range, 1.7–2.8] million cases) to 96 (range, 79–114) per 100 000 population (1.8 [range, 1.5–2.2] million cases) in 2018. The estimated number of TB deaths more than halved in the same period, from 209 000 (range, 178 000–242 000) (12 [range, 10–14] deaths per 100 000 population) in 2000 to 90 000 (4.7 [4.3–5.1] deaths per 100 000 population) in 2018 (**Fig. 1**). Since 2015, when the End TB Strategy and the Regional Framework: 2016–2020 were endorsed, the estimated incidence rate and number of TB deaths have decreased by 3% and 10%, with annual reductions of 1.0% and 3.4%, respectively. The estimated TB incidence and mortality rates among people living with HIV (PLHIV) have both remained low in the Region (2.1 [range, 1.5–2.8] and 0.34 [range, 0.25–0.43] per 100 000 population in 2018, respectively).

[**insert** Figure 1]

The decreasing trends in TB incidence and mortality observed in the Region are broadly in line with global trends and are driven mainly by improvements in TB control in China. When data from China are excluded, the estimated TB incidence rate in 2018 doubles to 196 (range, 121–292) cases per 100 000 population. The estimated TB incidence rate was lower in the subregion of the Pacific island countries than in other parts of the Region, but there has been no decrease in incidence since 2000, the rate ranging from 54 (range, 43–66) per 100 000 population in 2002 to 75 (range, 58–94) per 100 000 population in 2016.

**Figure 2:**
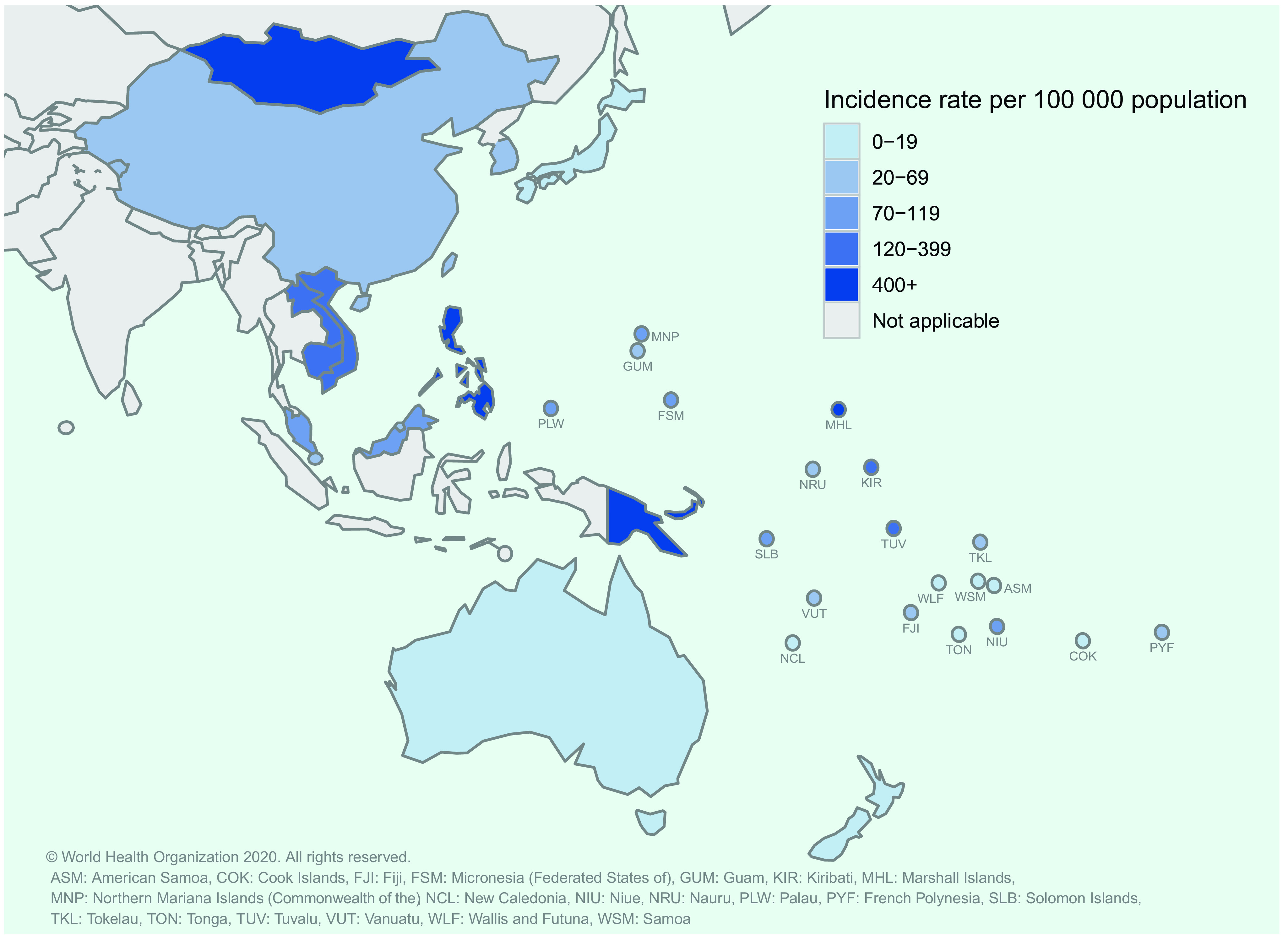
Estimated TB incidence rates per 100 000 population in countries and areas in the Western Pacific Region, 2018

**Figure 3:**
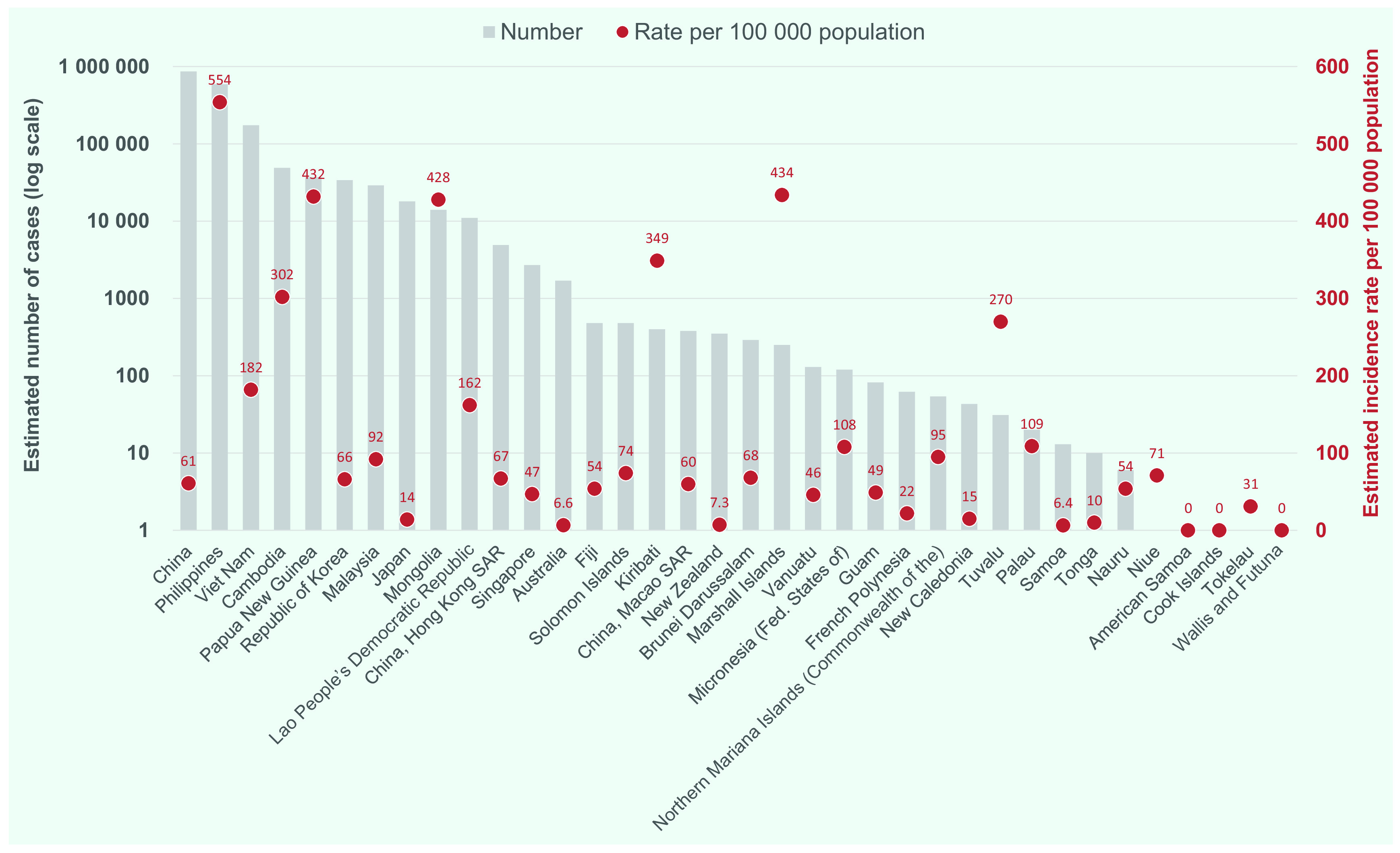
Estimated numbers of incident TB cases and TB incidence rates per 100 000 population in countries and areas in the Western Pacific Region, 2018

The estimated incidence of TB varies widely among countries in the Region (**Fig. 2**). In 2018, nearly 80% of all estimated TB cases occurred in just two countries, China (47% or 866 000 [range, 740 000–1 000 000] cases) and the Philippines (32% or 591 000 [range,  332 000–924 000] cases). A further 9% (174 000 [range, 111 000–251 000] cases) occurred in Viet Nam. In 2018, six countries had an estimated TB incidence rate of more than 300 cases per 100 000 population. The highest incidence rate was recorded in the Philippines (554 [range, 311–866] cases per 100 000), followed by the Marshall Islands (434 [range, 332–549] cases per  100 000), Papua New Guinea (432 [range, 352–521] cases per 100 000), Mongolia (428 [range, 220–703] cases per 100 000), Kiribati (349 [267–441] cases per  100 000) and Cambodia (302 [range, 169–473] cases per 100 000) (**Fig. 3**). Six countries and areas, American Samoa, Australia, Cook Islands, New Zealand, Samoa and Wallis and Futuna, had an estimated TB incidence rate of < 10 cases per 100 000 population in 2018.

[**insert** Figure 2]

[**insert** Figure 3]

### TB case notifications

**Figure 4:**
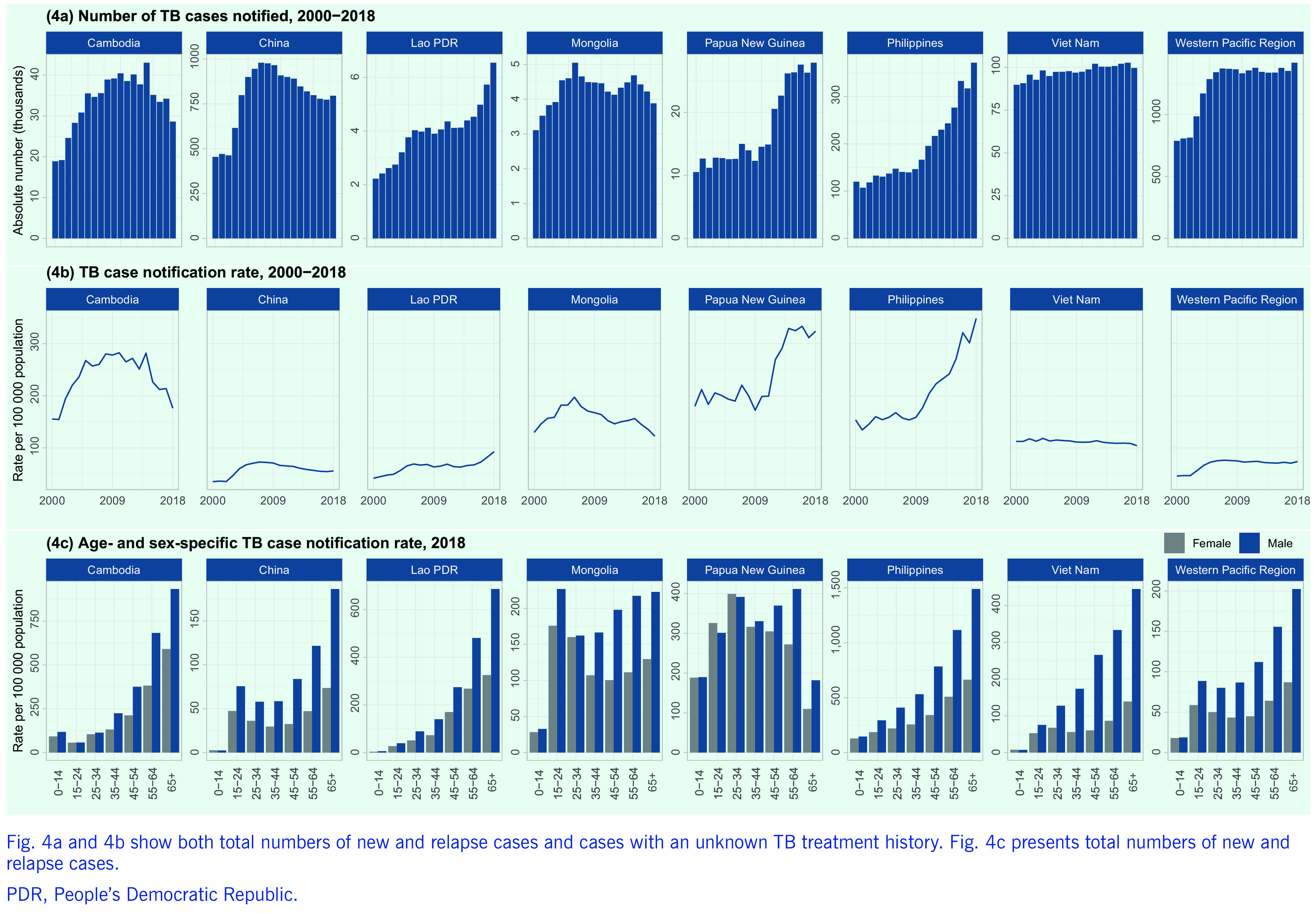
Case notifications of all forms of TB in the Western Pacific Region and in the seven focus countries, 2000–2018

The number of case notifications in the Region rose sharply between 2000 and 2007, mainly reflecting increased reporting from China, but has since remained stable, with 1 416 729 new and relapse cases notified in 2018 (a case notification rate of 74 per 100 000 population) (**Fig. 4a**). Trends in case notification from countries vary widely. During the past decade in the seven focus countries, decreasing case notification rates were observed in Cambodia, China and Mongolia, and increasing rates were reported in the Lao People's Democratic Republic, Papua New Guinea and the Philippines (**Fig. 4b**).

[**insert** Figure 4]

In the Region, the highest TB notification rate was for males aged (*3*)65 years (202 cases per 100 000 population), with a general tendency to higher case notification rates for older age groups (**Fig. 4c**). The exceptions were Mongolia and Papua New Guinea, where the proportions of younger people (0–24 years) among total cases were still high (32% and 46%, respectively), suggesting high rates of transmission in the general community. The male-to-female ratio of TB cases was high in adults (from 1.5 in those aged 15–24 years to 2.5 in those aged 45–54 years), with the largest differences being observed in older groups.

### Drug-resistant TB

Between 2015 and 2018, the numbers of laboratory-confirmed MDR/RR-TB cases and of patients enrolled in second-line treatment increased by 50% and 47%, respectively. During the same period, drug susceptibility testing (DST) coverage of bacteriologically confirmed cases rose from 28% in 2015 to 51% in 2018 but still remains far below the 100% target.

**Figure 5:**
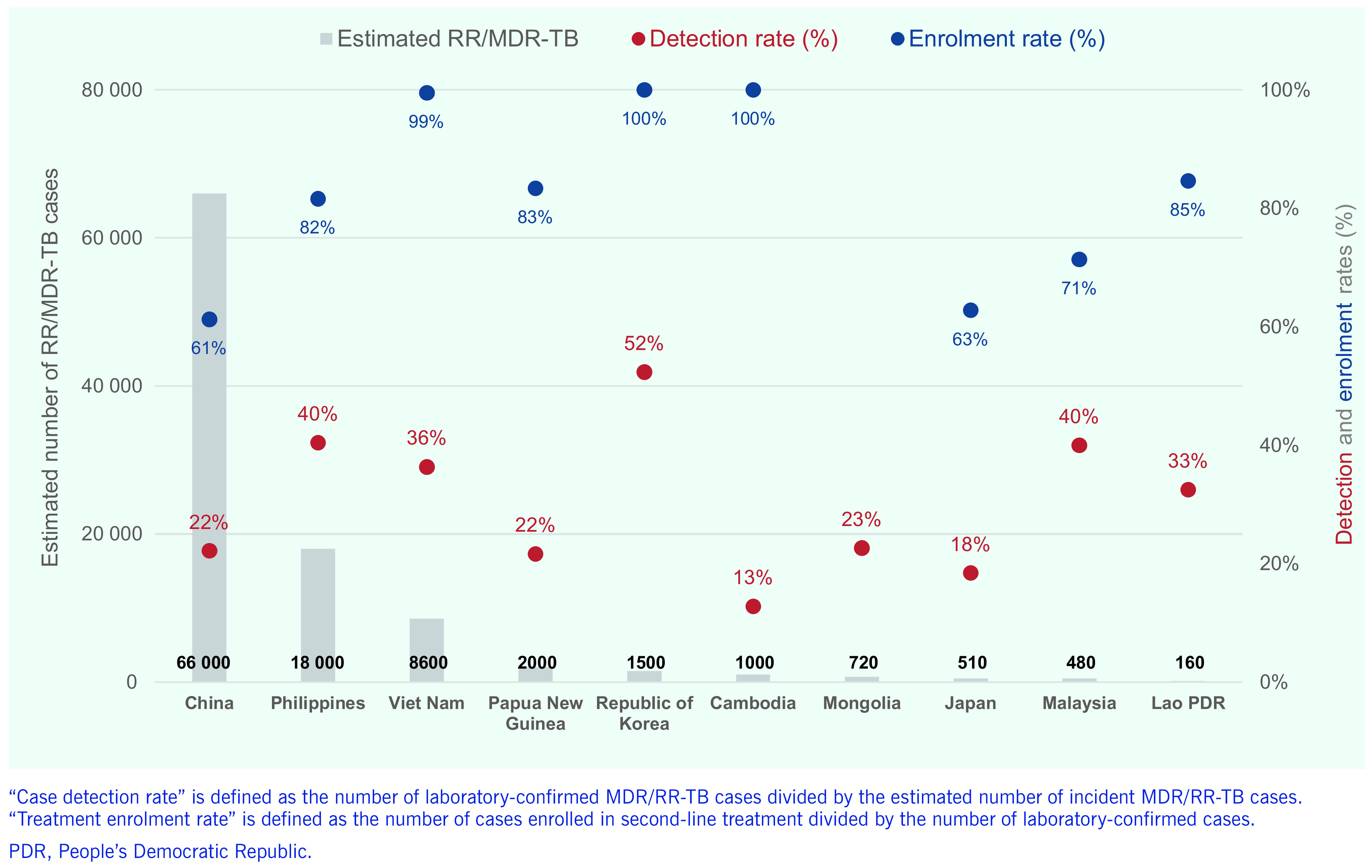
Estimated numbers of MDR/RR-TB incidence and detection and treatment enrolment rates for MDR/RR-TB in the 10 most-affected countries in the Western Pacific Region, 2018

China had the highest estimated number of MDR/RR-TB cases (66 000 [range, 50 000–85 000]) in 2018, followed by the Philippines (*n* = 18 000 [range, 7700– 32 000]), Viet Nam (*n* = 8600 [range, 5400–13 000]), Papua New Guinea (*n* = 2000 [range, 1200–2900]), the Republic of Korea (*n* = 1500 [range, 1300–1700]), Cambodia (*n* = 1000 [range, 460–1900]), Mongolia (*n* = 720 [range, 340–1200]), Japan (*n* = 510 [range, 220–930]), Malaysia (*n* = 480 [range, 360–620]) and the Lao People's Democratic Republic (*n* = 160 [range, 65–280]) (**Fig. 5**). These 10 countries accounted for more than 99% of the total estimated MDR/RR-TB case load in 2018 (*n* = 99 000). Importantly, case detection rates remained low in all these countries, ranging from 13% in Cambodia to 52% in the Republic of Korea. The rates of enrolment in treatment after diagnosis were excellent in Cambodia (100%), the Republic of Korea (100%) and Viet Nam (99%) but suboptimal in China (61%), Japan (63%) and Malaysia (71%).

[**insert** Figure 5]

### Indicators of collaborative TB/HIV activities

**Figure 6:**
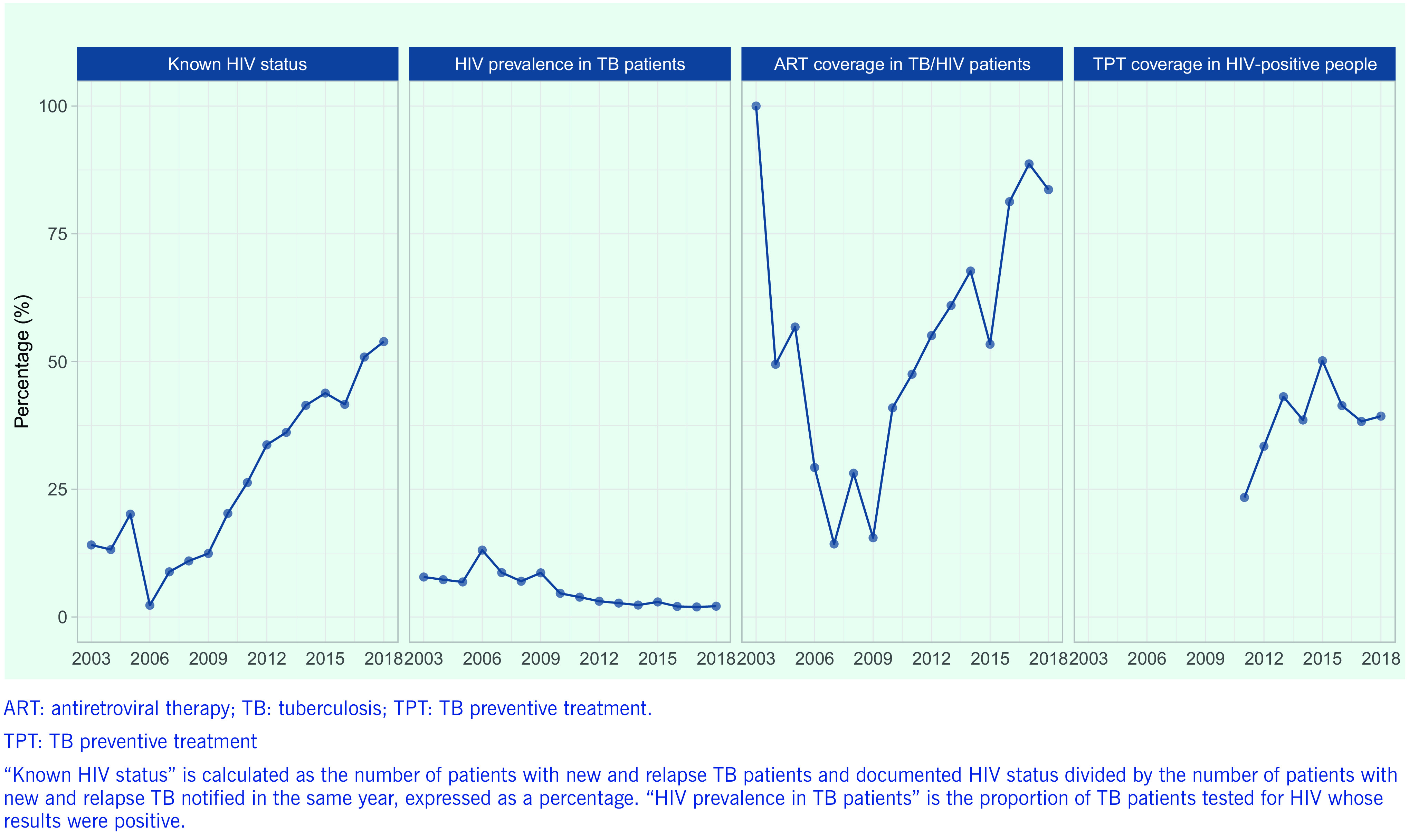
Trends in key indicators of collaborative TB/HIV activities in the Western Pacific Region, 2003–2018

Key indicators for TB-HIV care and collaborative activities have improved over time (**Fig. 6**). The proportion of TB cases with known HIV status increased substantially, from 12% in 2009 to 54% in 2018, although the proportion remains well below the target of 100% and the global average of 64%. The HIV prevalence among tested TB cases fell from a high of 13% in 2006 to < 3% in 2016, which has been maintained, reflecting more comprehensive screening. The proportion of TB/HIV co-infected patients receiving antiretroviral therapy (ART) has increased over time, reaching 84% in 2018 (based on reporting from 13 countries); however, this is also below the 100% target and does not include delays in initiation, as many countries still do not meet this reporting requirement. Coverage of TB preventive treatment (TPT) in PLHIV remains at < 50% (based on reports from only eight countries).

[**insert** Figure 6]

### Treatment outcomes

**Figure 7:**
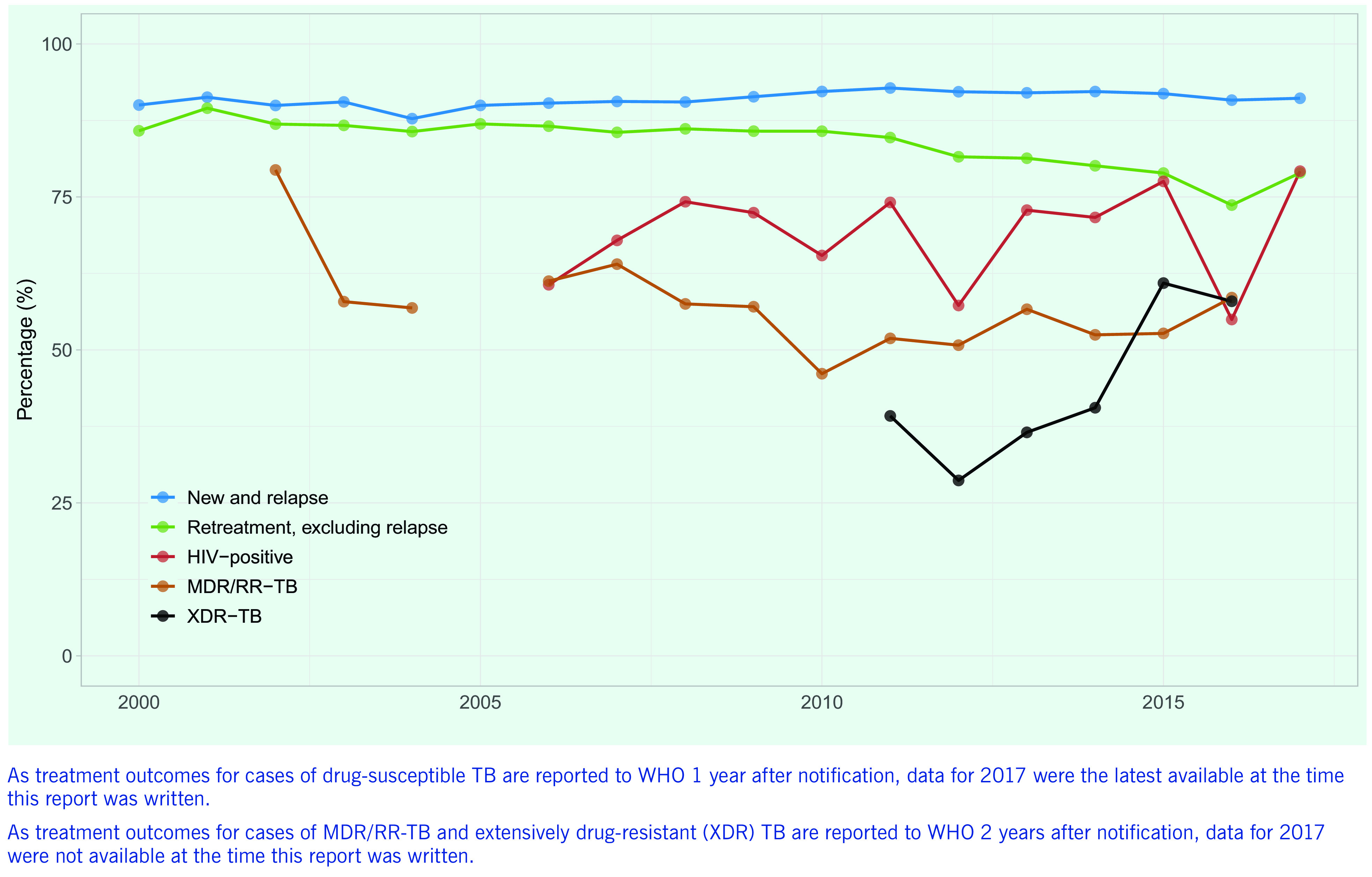
Trends in TB treatment success rates for different patient categories in the Western Pacific Region, 2000–2017

**Figure 8:**
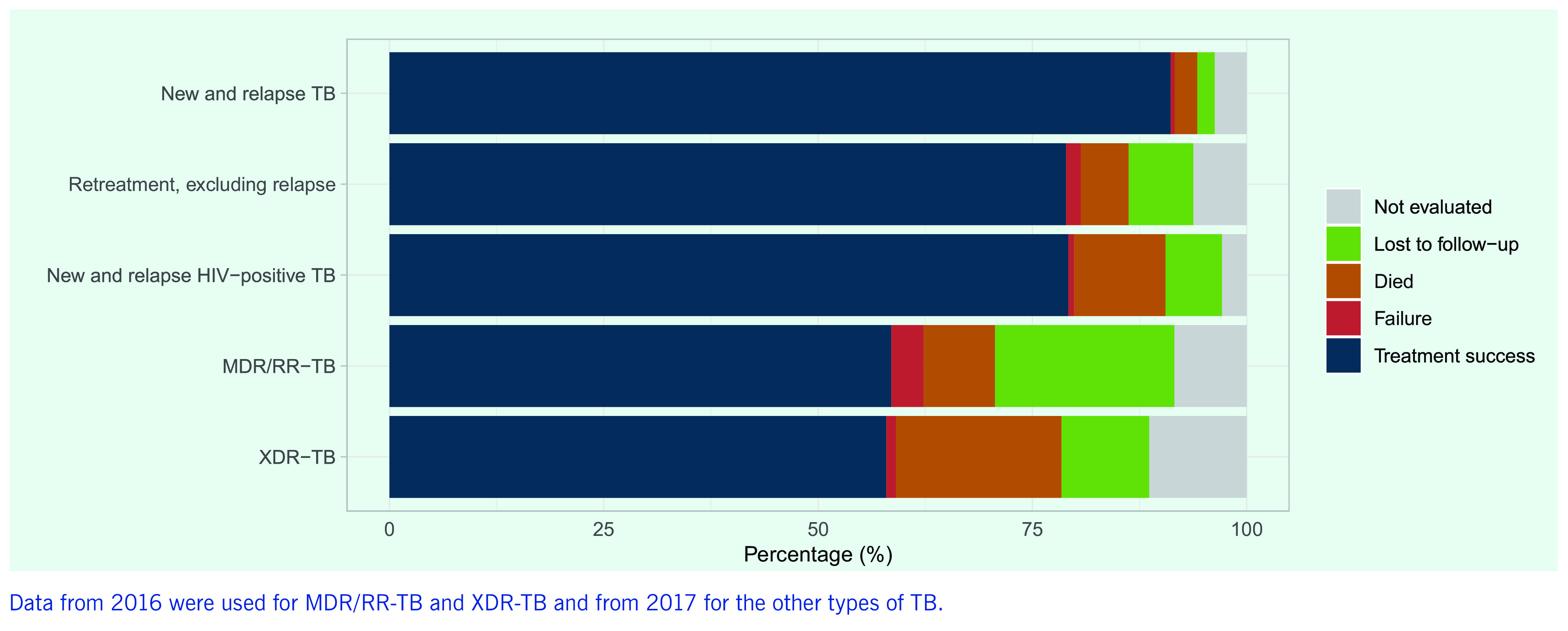
TB treatment outcomes by patient category in the Western Pacific Region, 2018

A TB treatment success rate (new and relapse cases) of > 90% has been maintained at regional level for over a decade (**Fig. 7**). The rate is due mainly to high treatment success rates in a few countries with large TB caseloads, including China (93%) and Viet Nam (92%), which tends to hide poor rates in many smaller countries. Overall, 20 of 32 reporting countries and areas (> 60%) had treatment success rates of < 85%, and success rates of < 70% were reported by countries and areas such as Hong Kong Special Administrative Region SAR (China) and Japan, where TB predominantly affects the elderly, but also in some Pacific island countries such as Papua New Guinea and Tuvalu, which have younger populations. The treatment outcomes of patients with TB/HIV co-infection and MDR/RR-TB remain suboptimal in most countries and at regional level (**Fig. 8**), the proportions being 79% and 59%, respectively, in 2018 (reflecting the 2017 and the 2016 patient cohorts, respectively).

[**insert** Figure 7]

[**insert** Figure 8]

### TB care cascade

**Figure 9:**
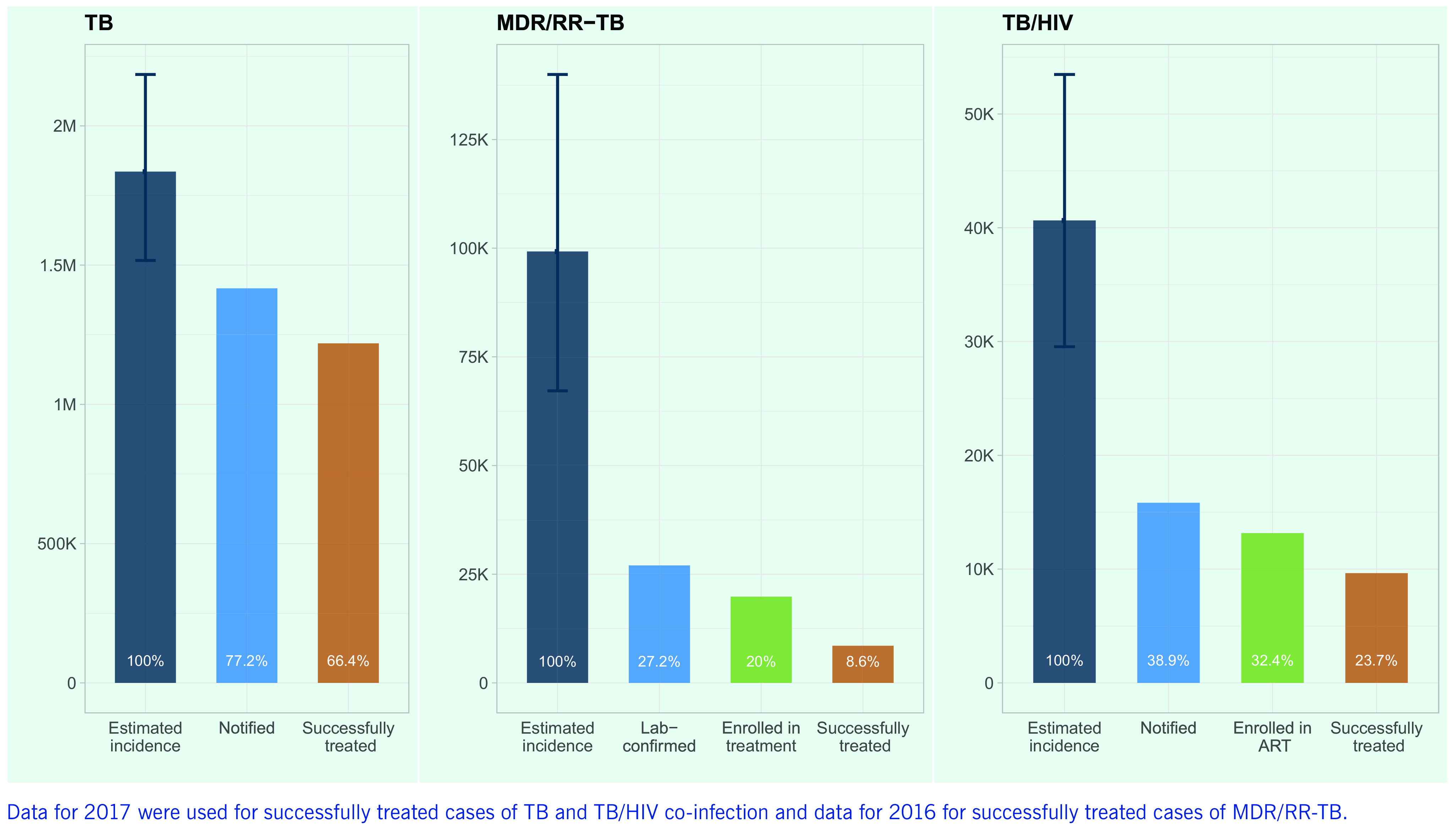
Key gaps in the cascade of care for TB, MDR/RR-TB and TB/HIV co-infection in the Western Pacific Region, 2018

**Fig. 9** shows gaps in the cascade of care for TB, DR-TB and TB/HIV co-infection in the Western Pacific Region. Of an estimated 1.8 million (range, 1.5–2.2 million) incident cases of TB in 2018, 5.4% (*n* = 99 228) were estimated to be MDR/RR-TB and 2.2% (*n* = 40 638) to be co-infected with HIV. Gaps in the TB care cascade in the Region remain substantial, especially for DR-TB and TB/HIV. Treatment coverage was relatively high for TB, at 77.2% (range, 64.9–93.4%), but low for TB/HIV co-infection (38.9%, range [29.6–53.6%]) and MDR/RR-TB (27.2%, range [19.3–40.2%]). Major gaps between the numbers of notified and confirmed MDR/RR-TB cases and patients enrolled in second-line TB treatment and in initiation of ART among HIV-positive TB patients are of particular concern. The proportions of estimated incident TB cases successfully treated for TB, MDR/RR-TB and TB/HIV co-infection were 66.4%, 8.6% and 23.7%, respectively.

[**insert** Figure 9]

### TB prevention

TPT coverage among PLHIV remained low, at 39%, in 2018. This figure is based on reports from eight countries, and coverage in non-reporting countries may be even lower. TPT coverage of children under 5 years who were household contacts of a bacteriologically confirmed case of pulmonary TB was estimated to be very low (12%) from data for 14 countries.

### Patient costs due to TB

Eight countries in the Region have conducted national TB patient cost surveys and have established a baseline from which to monitor progress towards elimination of catastrophic costs due to TB. In the surveys, 35–70% of TB-affected families reported facing catastrophic costs.

### Proposed “top-10” indicator scorecard

**Table 1** represents a proposed colour-coded scorecard of the “top-10” TB indicators of programme performance towards the End TB Strategy targets. In 2018, treatment coverage remained low (< 60%) in some countries with a high TB burden (Cambodia, Lao People's Democratic Republic, Mongolia and Viet Nam), and low treatment success rates were reported in Japan (68%), Hong Kong Special Administrative Region SAR (China) (65%) and some Pacific island countries, including Papua New Guinea (68%) and Tuvalu (68%). The proportion of TB patients tested with a WHO-recommended rapid diagnostic test (molecular techniques to detect TB among people with signs or symptoms of TB, such as Xpert MTB/RIF®) at the time of diagnosis remained low in many countries (11 countries reported < 60%). DST coverage was extremely low (< 5% of bacteriologically confirmed TB cases) in Cambodia, the Philippines, Papua New Guinea and Solomon Islands, while many with successful Xpert MTB/RIF® roll-out programmes reported high coverage. Despite a long-standing policy to test all TB patients for HIV infection, the proportion of TB patients of known HIV status remained low in many countries. The case fatality ratios were high in Japan (16%), Lao People's Democratic Republic (22%), Papua New Guinea (13%) and Vanuatu (17%). Contact investigation coverage and treatment coverage for new drugs were among the “top-10” TB indicators; however, data are not available. The 2020 End TB Strategy milestones of reduced TB incidence rate and deaths were achieved by 2018 by only 19% (7/36) and 11% (4/36) of the countries in the Region, respectively.

**Table 1:** Proposed scorecard for accessing the “top-10” indicators for monitoring implementation of the End TB Strategyin the Western Pacific Region

	Top Indicators (%)									% Change from 2015	
	Treatment coverage	Treatment success rate	TB-affected households with catastrophic costs due to TB	TB patients tested using WRD at diagnosis	LTBI treatment coverage (PLHIV)	LTBI treatment coverage (Child contact)	DST coverage for TB patients	Documentation of HIV status	Case fatality ratio	Estimated incidence rate	Estimated deaths
**Recommendeed Target†**	**^3^90%**	**^3^90%**	**0%**	**^3^90%**	**^3^90%**	**^3^90%**	**100%**	**100%**	**£5%**	**-20%**	**-35%**
American Samoa*	**-**	**80**	**-**		**-**	**-**	**100**	**-**	**-**	**-**	**-**
Australia	**87**	**82**	**-**	**-**	**-**	**-**	**93**	**88**	**3**	**10**	**-3**
Brunei Darussalam*	**87**	**75**	**-**	**-**	**0**	**0**	**100**	**100**	**6**	**15**	**-4**
China	**92**	**93**	**-**	**15**	**-**	**-**	**63**	**60**	**5**	**-6**	**-5**
Cook Islands*	**-**	**-**	**-**	**-**	**-**	**-**	**-**	**-**	**-**	**-**	**-**
Fiji*	**80**	**81**	**40**	**95**	**-**	**100**	**109**	**89**	**9**	**4**	**11**
Federated States of Micronesia (Federated States of)*	**80**	**88**	**-**	**-**	**-**	**-**	**86**	**0**	**11**	**-10**	**-7**
Guam*	**87**	**89**	**-**	**69**	**-**	**-**	**100**	**96**	**8**	**-9**	**-7**
China, Hong Kong Special Administrative Region SAR	**87**	**65**	**-**	**32**	**-**	**11**	**91**	**78**	**3**	**-7**	**-13**
Japan	**87**	**68**	**-**	**-**	**-**	**62**	**74**	**8.3**	**16**	**-14**	**-8**
Cambodia	**58**	**94**	**-**	**-**	**-**	**-**	**0**	**94**	**7**	**-18**	**-9**
Kiribati*	**80**	**89**	**-**	**50**	**-**	**-**	**100**	**51**	**11**	**-38**	**1**
Republic of Korea	**94**	**83**	**-**	**26**	**-**	**60**	**84**	**-**	**7**	**-17**	**-9**
Lao People's Democratic Republic	**57**	**89**	**63**	**63**	**-**	**18**	**52**	**81**	**22**	**-11**	**-33**
China, Macao SAR*	**87**	**82**	**-**	**68**	**-**	**20**	**99**	**92**	**8**	**-16**	**-12**
Marshall Islands*	**170**	**83**	**-**	**77**	**-**	**-**	**100**	**22**	**11**	**45**	**47**
Mongolia	**29**	**91**	**70**	**39**	**0**	**7.4**	**79**	**70**	**3**	**0**	**-1**
Northern Mariana Islands (Commonwealth of the)*	**87**	**98**	**-**	**32**	**-**	**-**	**100**	**100**	**8**	**65**	**64**
Malaysia	**87**	**81**	**-**	**-**	**38**	**88**	**78**	**82**	**5**	**3**	**1**
New Caledonia*	**87**	**35**	**-**	**5.4**	**-**	**-**	**100**	**30**	**8**	**-36**	**-34**
Niue*	**87**	**-**	**-**	**-**	**-**	**-**	**-**	**0**	**8**	**-**	**-**
Nauru*	**87**	**78**	**-**	**-**	**-**	**-**	**0**	**0**	**8**	**-51**	**-50**
New Zealand*	**87**	**82**	**-**	**-**	**-**	**100**	**0**	**0.3**	**4**	**1**	**3**
Philippines	**63**	**91**	**35**	**36**	**52**	**9.4**	**4**	**27**	**5**	**1**	**-8**
Palau*	**87**	**80**	**-**	**88**	**-**	**-**	**100**	**94**	**8**	**20**	**21**
Papua New Guinea	**75**	**68**	**54**	**-**	**21**	**27**	**0**	**55**	**13**	**0**	**12**
French Polynesia*	**87**	**81**	**-**	**63**	**-**	**-**	**100**	**85**	**8**	**15**	**17**
Singapore	**87**	**79**	**-**	**60**	**0**	**100**	**98**	**89**	**2**	**5**	**-11**
Solomon Islands*	**80**	**92**	**-**	**27**	**-**	**-**	**0**	**28**	**11**	**-14**	**-7**
Tokelau*	**-**	**-**	**-**	**-**	**-**	**-**	**-**	**-**	**11**	**-85**	**-84**
Tonga*	**87**	**82**	**-**	**89**	**-**	**-**	**100**	**100**	**8**	**-37**	**-36**
Tuvalu*	**87**	**68**	**-**	**74**	**-**	**-**	**89**	**100**	**8**	**30**	**35**
Viet Nam	**57**	**92**	**63**	**20**	**39**	**22**	**84**	**85**	**8**	**-9**	**-24**
Vanuatu*	**67**	**96**	**-**	**46**	**-**	**-**	**100**	**69**	**17**	**-27**	**17**
Wallis and Futuna*	**-**	**-**	**-**	**-**	**-**	**-**	**-**	**-**	**-**	**-**	**-**
Samoa*	**87**	**57**	**-**	**-**	**0**	**-**	**0**	**100**	**8**	**-43**	**-42**

Cutoff values for colour code											
**Green**	**^3^85**	**^3^85**	**£29**	**^3^80**	**^3^70**	**^3^70**	**^3^80**	**^3^85**	**£5**	** < 0**	** < 0**
**Yellow**	**60–84**	**75–84**	**30–59**	**60–79**	**50–69**	**50–69**	**60–79**	**75–84**	**6–9**	**0–5**	**0–5**
**Red**	**£59**	**£74**	**^3^60**	**£59**	**£49**	**£49**	**£59**	**£74**	**^3^10**	**^3^6**	**^3^6**

## Discussion

This epidemiological analysis shows regional progress over time in certain programmatic areas, including sustained, good treatment outcomes, improvements in TB/HIV indicators and improved case detection and enrolment of MDR/RR-TB cases. Programmes should continue to extend diagnosis and case finding, enhance service quality and increase resources for TB programmes.

The number of sites in the Region that provide TB diagnoses with Xpert MTB/RIF® increased by 48%, from 1351 in 2015 to 1998 sites in 2018, based on reports from 15 countries and areas. (*4*) Increased case notification rates were reported in several high-burden countries (Lao People's Democratic Republic, Papua New Guinea and Philippines), which may reflect intensified case detection in these countries. Careful monitoring will be necessary to ensure that the numbers do not decrease over time. The same observation applies to Pacific island nations, such as the Marshall Islands, where active case finding projects may transiently increase the case numbers; if the projects are successful, they should be followed by drastic reductions in case numbers.

Between 2015 and 2019, total funding, both domestic and international, for TB in countries and areas of the Region increased by 67%, from US$ 504 million to US$ 843 million, although the funding gap remains large at US$ 249 million (23%). (*3*, *4*) Indications of increased resource allocation and service provision are encouraging, as they may reflect increased political commitment from governments in the Region.

Overall progress in reducing the TB burden in the Western Pacific Region is slow, as little progress has been made in some countries. In 2018, the TB incidence rate was 96 per 100 000 population, whereas the 2020 milestone is 79 per 100 000 population, and an estimated 97 000 deaths from TB occurred, whereas the 2020 milestone is 70 200. In view of the current annual reductions in the TB incidence rate (1.0%) and the number of deaths (3.4%), the Region is unlikely to achieve the 2020 milestones and other targets of the End TB Strategy.

Our analyses signal several challenges for the Region: (1) wide variation among countries in the geographical distribution and incidence of TB, including the fact that TB continues to largely affect younger age groups in several countries; (2) a sizeable proportion of TB cases remain unreached, undiagnosed or unreported; (3) insufficient coverage of DST and use of WHO-recommended rapid diagnostic tests; (4) suboptimal TB treatment success rates in some countries and poor treatment outcomes for PLHIV and patients with DR-TB; (5) limited TPT coverage of PLHIV and child contacts; and (6) a substantial proportion of TB-affected families facing catastrophic costs.

The wide variation in TB epidemiology and contextual factors among countries poses a challenge for mounting a coordinated regional TB response. In countries with a low TB burden, such as Australia and New Zealand, > 80% of the cases notified are in foreign-born individuals, and TB is essentially an imported disease. (*4*) In countries and areas with ageing populations, such as Japan and the Republic of Korea, TB occurs mainly in the elderly, people aged (*3*)65 years accounting for 66.7% of total case notifications in Japan, 45.4% in the Republic of Korea and 43.7% in Hong Kong Special Administrative Region SAR (China) in 2018. (*4*) In lower-income high-burden countries, undernutrition is considered a major risk factor for TB, (*1*) and high rates of cigarette smoking may contribute to over-representation of TB in men. (*9*) In Pacific island countries, diabetes is highly prevalent and considered a major driver of the TB epidemic. (*10*, *11*) Understanding of population-level risk factors for TB by analysis of routine surveillance data, survey results and facility records is important to design targeted interventions. The Western Pacific Region therefore requires a tailored regional strategy to guide the response in various epidemiological and contextual settings, including for small Pacific island countries with unique geographical challenges and high TB incidence rates per capita, such as Kiribati, Marshall Islands and Tuvalu.

TB case notifications are affected by many factors, and careful analysis and interpretation are required to understand the strengths and weaknesses of national TB programmes. Interventions such as community-based active case finding, facility-based systematic screening, increased use of sensitive screening and diagnostic tools and algorithms, improved referral mechanisms and specimen transport, engagement of private and other health sectors and mandatory notification policies can increase case notifications. (*12*, *13*) Although decreasing numbers of case notifications may represent a true decrease in TB incidence, they may be due to decreased case finding or reduced TB programme funding and functioning. (*12*) In the Region, significant funding for TB is provided by the Global Fund to Fight AIDS, Tuberculosis and Malaria. Over time, such funding has supported expansion of TB services and improved TB surveillance in many high-burden countries, including Cambodia, China, the Lao People's Democratic Republic, Mongolia and Papua New Guinea. (*14*) In the Philippines, over-reliance on chest X-ray for diagnosis resulted in increased case notifications of clinically diagnosed TB in the 2010s. Subsequently, the introduction of a mandatory TB notification policy increased reporting from the private sector, resulting in a sharp rise in case notifications in 2018, although a national survey demonstrated many unidentified cases. (Epidemiological review for tuberculosis in the Philippines. 2019, unpublished). The recent increase in TB case notifications in the Lao People's Democratic Republic can be attributed to intensified case-finding among high-risk populations (Epidemiological review for tuberculosis in Lao People's Democratic Republic. 2019, unpublished), and a decrease in case notifications in Cambodia is probably attributable to reduced community-based case-finding activities because of reduced external funding (Epidemiological review for tuberculosis in Cambodia. 2019, unpublished). Trends in case notification should therefore be considered carefully in relation to any major programmatic changes. In most instances, an emphasis on “finding missing cases” is appropriate. Furthermore, given the geographical variation in TB epidemiology within a country, monitoring and evaluation should be strengthened at subnational level to ensure better-targeted interventions guided by data. (*15*)

Accurate diagnosis is a fundamental component of TB care. Rapid molecular diagnostics ensure early detection and prompt treatment, while testing for drug resistance is essential to ensure appropriate treatment. (*1*) As part of TB laboratory-strengthening in the End TB Strategy, countries are encouraged to adopt a policy to use a WHO-recommended rapid diagnostic test as the initial test for all people with presumptive TB and to provide universal access to DST for patients with bacteriologically confirmed TB. (*1*) In the Region in 2018, only 25% of countries and areas (*n* = 9/36) had a policy to use a WHO-recommended rapid test at diagnosis, and only 39% (*n* = 14/36) had a policy of universal access to DST. (*4*) This explains the insufficient DST coverage and use of WHO-recommended rapid diagnostic tests that we observed. Adoption and implementation of such policies requires substantial investment, with sustainable financing for TB laboratory services. This remains a major challenge, but successful examples exist in other parts of the world (*16*) to guide implementation. In-depth analysis of national networks for TB diagnosis and specimen transport to understand the levels of underutilization or access to existing diagnostic tools and investigation of opportunities for collaboration with other public health programmes and the private sector could pave the way for a massive roll-out of WHO-recommended rapid diagnostic tests and expanded DST. This will be essential to close the gap in case detection and improve treatment outcomes for patients with MDR/RR-TB. It could also reduce MDR/RR-TB transmission by prompt initiation of effective treatment. Estimating longer-term population-level benefits of such policies could convince policy-makers to take action.

Treatment outcomes remain a challenge in many countries in the Region, particularly for patients with DR-TB and TB/HIV co-infection. WHO recommends a wide range of interventions to facilitate early diagnosis and optimal treatment, including screening of PLHIV for TB, early initiation of ART, better infection control, provision of TPT, wider use of more effective MDR-TB treatment regimens, active TB drug-safety monitoring and management and more patient-centred models of care. (*1*) Digital technologies to support adherence to TB medication are becoming increasingly available. (*17*) Assessing and addressing gaps in such interventions and promoting the uptake of new tools and innovations should help to improve treatment outcomes. Furthermore, risk groups and geographical areas in which poor treatment outcomes are reported should be identified to guide the most appropriate targeted responses. Generally, treatment outcomes vary by geographical areas according to the local TB epidemiology and response. (*18*) (Epidemiological review for tuberculosis in the Philippines. 2019, unpublished; Epidemiological review for tuberculosis in Lao People's Democratic Republic. 2019, unpublished). In Japan, the overall treatment success rate is low mainly because of a high mortality rate among the elderly, the population most affected by TB. (*19*) Other countries in which the population is ageing rapidly and the proportion of TB cases among the elderly is increasing may face a similar challenge in the future. (*20*) Global and regional TB programmes should be ready to address the issue of TB among the elderly on the basis of evidence-based guidance and effective interventions. (*21*)

TPT is an essential intervention for achieving the goals of the End TB Strategy. The first United Nations high-level meeting on TB, held in 2018, set a new global target, to provide TPT to at least 30 million people in the period 2018–2022, comprising 6 million PLHIV, 4 million children under 5 years who are household contacts of people affected by TB and 20 million other household contacts of TB cases. (*1*) At the current rate of treatment enrolment, the world will not reach the target for household contacts, and, in May 2020, global TB partners released a joint call to action to scale up access to TPT. (*22*) In view of the low coverage of TPT in the Region, rapid adoption and implementation of the recently published TPT guidelines (*23*) is critical. In particular, a systems approach is necessary to make contact investigation and TPT integral parts of primary health care services as an essential public health function. (*24*)

TB patients continue to bear a heavy financial burden, despite the provision of free TB services in most countries in the Region. National surveys of the cost of TB patients have provided solid evidence that TB-affected families face severe financial hardship, non-medical costs and income loss due to TB are major components, poor households get poorer due to TB and loss of jobs, and households of patients with DR-TB and TB/HIV co-infection face significantly higher costs. (*1*, *25*) These results are powerful arguments for initiating policy dialogue with other programmes and sectors and as a basis for policies and strategies to optimize health care delivery and financing and increase social protection for TB patients. (*1*)

This analysis has several limitations. First, data for several indicators, such as TPT and DST coverage, were not complete, and therefore the regional averages are not always from all countries. Second, regional averages of the indicators and estimated burden are driven largely by the numbers recorded in China and the Philippines, where the numbers of cases are relatively large; therefore, the findings must be interpreted carefully. Third, the number and proportion of successfully treated patients used in the analysis of the care cascade were for patient cohorts in previous years (2017 for TB and TB/HIV, 2016 for DR-TB); therefore, the gaps calculated for 2018 may not be accurate, although we believe them to be very close approximations of the complete data set. Finally, in countries and areas with few cases, the percentage changes in estimated incidence and mortality shown on the scorecard may not reflect true trends because of possible large fluctuations. Despite these limitations, our analysis provides comprehensive, useful insights into the regional TB situation based on several years of data reported according to adopted, well established case definitions from nearly all the countries in the Region.

If the Region is to achieve the End TB Strategy targets beyond the interim 2020 milestones, it must overcome several challenges. Some of these challenges lie outside national TB programmes and even the health sector, requiring a multisectoral response. (*3*) In addition, the COVID-19 pandemic has disrupted health services worldwide and poses a considerable challenge for TB programmes and for TB patients; however, it also provides an opportunity to increase investment in health and to promote multisectoral responses to health system transformation, from which the TB programme can benefit and to which it can contribute. The WHO Regional Office for the Western Pacific will continue to provide data-driven, evidence-based regional guidance and will support Member States on their journey towards ending TB.
